# Deep Learning Based on B-Mode and Color Doppler Ultrasound for Differentiation of Primary Thyroid Lymphoma and Hashimoto’s Thyroiditis: A Retrospective Single-Center Study

**DOI:** 10.3390/diagnostics16121909

**Published:** 2026-06-19

**Authors:** Juanmei Chen, Zijian Deng, Yong Chen, Ruiheng Ye, Jiawu Li, Yi Tao, Buyun Ma, Yushuang He

**Affiliations:** 1 Department of Ultrasound, West China Hospital, Sichuan University, Chengdu 610041, China; chenjuanmei@stu.scu.edu.cn (J.C.);; 2 School of Computing Science, Sichuan University, Chengdu 610022, China; dengzijian@stu.scu.edu.cn; 3 Department of Ultrasound Medicine, Sun Yat-sen Memorial Hospital, Sun Yat-sen University, Guangzhou 510120, China

**Keywords:** primary thyroid lymphoma, diffuse large B-cell lymphoma, mucosa-associated lymphoid tissue lymphoma, Hashimoto’s thyroiditis, ultrasound, deep learning, image-level classification

## Abstract

**Background/Objectives**: Primary thyroid lymphoma (PTL), including diffuse large B-cell lymphoma (DLBCL) and mucosa-associated lymphoid tissue (MALT) lymphoma, share substantial overlap in ultrasound appearance with Hashimoto’s thyroiditis (HT), making preoperative differentiation challenging. This study aims to develop and validate a deep learning model based on B-mode ultrasound (BMUS) and color Doppler ultrasound (CDUS) for image-level differentiation of DLBCL, MALT lymphoma, and HT. **Methods**: This retrospective single-center study included 1294 ultrasound images from 290 patients (313 lesions) who underwent preoperative ultrasound examination at West China Hospital between September 2002 and September 2024. All images from the same lesion were assigned to the same data partition, and the dataset was split at the lesion level into training and test sets at an 8:2 ratio. A Frequency-Adaptive WT-ResNet model incorporating wavelet transform convolution and a frequency-adaptive gating mechanism was developed. The primary analysis was performed at the image level. The performance of the model was compared with that of three ultrasound physicians with different levels of experience. Grad-CAM was used for visual interpretation. An exploratory external validation was performed using an independent dataset from Sun Yat-sen Memorial Hospital. **Results**: In the test set, the model achieved a macro-average AUC of 0.927 (95% CI: 0.889–0.960), with class-specific AUCs of 0.899 for DLBCL, 0.946 for MALT lymphoma, and 0.937 for HT. The macro-average balanced accuracy was 0.866, compared with 0.827 for that of the best-performing senior physician. The exploratory validation set yielded a macro-average AUC of 0.796 (95% CI: 0.686–0.888), with class-specific AUCs of 0.806 for DLBCL, 0.825 for HT, and 0.756 for MALT lymphoma. Grad-CAM showed that the model focused on lesion-internal echotexture and lesion-transition regions with class-dependent patterns. **Conclusions**: A deep learning model based on BMUS and CDUS showed promising performance for image-level differentiation of DLBCL, MALT lymphoma and HT in a single-center retrospective cohort. The model outperformed three ultrasound physicians and may serve as a potential decision-support tool. However, the exploratory external validation results should be interpreted as preliminary, and larger multicenter cohorts remain necessary to confirm model generalizability.

## 1. Introduction

Primary thyroid lymphoma (PTL) is a rare thyroid malignancy, accounting for approximately 5% of all thyroid malignancies, with diffuse large B-cell lymphoma (DLBCL) and mucosa-associated lymphoid tissue (MALT) lymphoma as the predominant subtypes [1,2]. Hashimoto’s thyroiditis (HT) is a well-established risk factor for PTL, and a substantial proportion of patients with PTL have coexisting HT [3].

Ultrasound is the first-line imaging modality for evaluating thyroid diseases. However, PTL frequently arises in the background of HT, and these two conditions share overlapping ultrasound features, including diffuse thyroid enlargement, marked hypoechoicgenicity and heterogeneous echo texture, making differential diagnosis challenging [4,5,6]. Ultrasound-guided biopsy is essential for definitive diagnosis [7]. However, fine-needle aspiration (FNA) has limited sensitivity for lymphoma and is often insufficient for subtype classification [8]. Core-needle biopsy (CNB) is invasive with bleeding risks and often requires supplementary molecular testing to achieve an accurate diagnosis [9,10]. Given that the prognosis of PTL is highly dependent on pathological classification and disease stage [11], there is a clinical need for noninvasive tools that can improve the preoperative differentiation of PTL and HT on ultrasound.

In recent years, artificial intelligence and deep learning have shown great potential in medical image analysis and tumor classification [12,13,14]. Previous studies have developed EfficientNet model utilizing H&E stained histopathological images for lymphoma subtype classification [15]. Multimodal LymphoMAP model integrating ^18^F-FDG PET imaging with clinical data was also developed to provide a robust tool for noninvasive diagnosis of lymphoma subtypes [16]. However, ultrasound offers notable advantages over pathology and ^18^F-FDG PET examination, including non-invasiveness, low-cost, wider availability and better repeatability. In addition, color Doppler ultrasound (CDUS) may provide complementary hemodynamic information beyond grayscale morphology, which could be particularly relevant for lesions with overlapping structural features [17]. Given the rarity of PTL and the complexity of its ultrasound appearance, a deep learning approach that leverages both B-mode ultrasound (BMUS) and CDUS may offer incremental diagnostic value.

In this study, we developed and evaluated a frequency-adaptive WT-ResNet deep learning model based on BMUS and CDUS images for the image-level differentiation of DLBCL, MALT lymphoma, and HT. We hypothesized that incorporating wavelet transform and a frequency-adaptive gating mechanism could improve discrimination among these entities by preserving diagnostically relevant fine-grained sonographic patterns. Our results showed that the proposed model achieved promising diagnostic performance in a single-center retrospective cohort and outperformed three ultrasound physicians in a blinded static-image comparison.

## 2. Materials and Methods

### 2.1. Study Design and Population

This retrospective single-center study was approved by the Institutional Review Board of the West China Hospital, Sichuan University, Chengdu, China (No. 2026292), and the requirement of individual consent for this retrospective analysis was waived. The patients who underwent preoperative thyroid ultrasound between September 2002 and September 2024 were consecutively enrolled. This study was reported with reference to the Checklist for TRIPOD+AI (2024) reporting guidance [18] (Supplementary Materials).

The inclusion criteria were (a) patients with PTL confirmed by histopathology with immunohistochemical analysis according to WHO classification criteria (based on CNB or surgical resection), with HT confirmed by histopathology using at least one method (based on FNA, CNB or surgical resection), (b) ultrasound examination completed prior to biopsy or surgery and (c) age ≥ 18 years. The exclusion criteria were (a) incomplete clinic or ultrasound data, (b) poor-quality ultrasound images unsuitable for analysis and (c) other concurrent thyroid malignancies.

**Figure 1:**
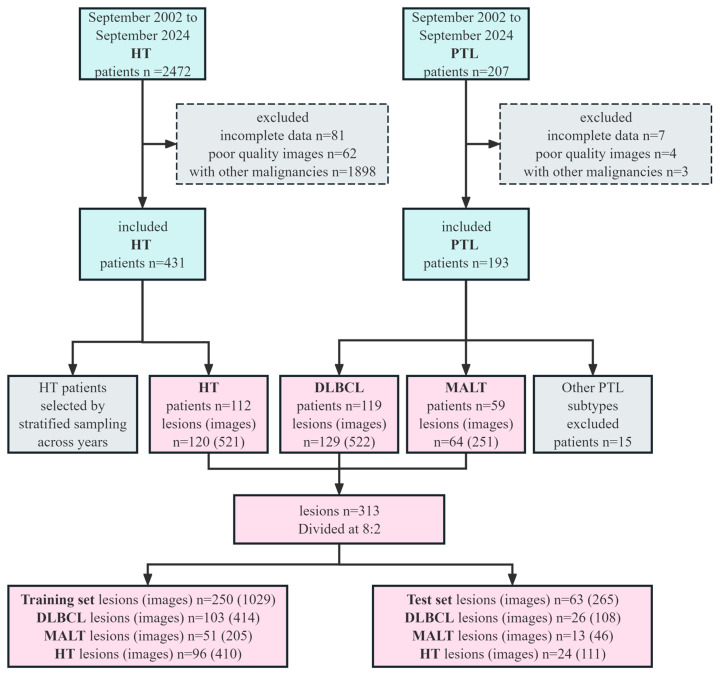
The flow chart of patient selection process. As both the model and the ultrasound physicians interpreted single static ultrasound images, the primary analysis was performed at the image level. All images from the same lesion were kept in the same data partition to avoid lesion-level data leakage.

A total of 313 lesions from 290 patients was included, comprising 129 DLBCL lesions from 119 patients, 64 MALT lymphoma lesions from 59 patients, and 120 HT lesions from 112 patients. In total, 1294 ultrasound images were analyzed, including 522 images for DLBCL, 251 images for MALT lymphoma, and 521 images for HT. For patients with pathologically confirmed lesions in both the left and right thyroid lobes, each lesion was treated as an independent lesion, but all images from the same lesion were assigned to the same data partition to avoid cross-partition data leakage. The dataset was randomly divided at the lesion level into training and test sets at a ratio of 8:2 using a random seed of 2025. The patient selection process is shown in Figure 1.

An exploratory external validation cohort was additionally collected from the Department of Ultrasound Medicine, Sun Yat-sen Memorial Hospital, between December 2015 and December 2025, including 14 DLBCL lesions with 43 images, 14 HT lesions with 42 images, and 3 MALT lymphoma lesions with 10 images. As the number of retrievable MALT lymphoma cases was limited, this dataset was used only for exploratory external validation.

### 2.2. Ultrasound Images Acquisition and Reader Comparison

Ultrasound images were acquired by multiple ultrasound systems (SonoScape, Samsung, Hitachi, Philips, GE, Siemens, Toshiba, ATL, and Mindray) equipped with 5–14 MHz linear-array transducer. All images were independently reviewed by two physicians with at least 5 years of experience in thyroid ultrasound, who were blinded to clinical information and pathological results. If no consensus was reached, discrepancies were resolved by the judgment of a senior radiologist with 20 years of experience in thyroid ultrasound. Clinical characteristics were extracted from electronic medical records system. The following ultrasound features were recorded for descriptive analysis: HT background (present or absent), lesion type (nodular, diffuse, or mixed) [19], abnormal cervical lymph nodes (present or absent), Adler grade (0–3) [20], boundary (distinct or indistinct), morphology (regular or irregular), aspect ratio (wider-than-tall, or taller-than-wide) and echogenicity (hypoechoic or marked hypoechoic). For HT, lesion type, boundary, morphology, and aspect ratio were not analyzed because HT usually involves diffuse parenchymal changes rather than an isolated measurable tumor boundary.

Static BMUS and CDUS images were exported in JPG format and underwent standardized preprocessing before model input. Each image was treated as an independent image-level sample after lesion-level partitioning. All images were resized to 224 × 224 pixels. Pixel intensities were converted to floating-point values and scaled to the range from 0 to 1. For BMUS images, intensity values were further normalized by per-image standardization to reduce acquisition-dependent contrast variation. For CDUS images, the three color channels were retained and normalized in the same way to preserve Doppler flow information. The same preprocessing pipeline was applied to training, test and validation images.

For reader comparison, three ultrasound physicians with senior, intermediate, and junior levels of experience independently evaluated the test images under blinded conditions. The readers independently reviewed the same BMUS and CDUS image set as the model on an image-by-image basis, and performed the same image-level three-class classification task. Clinical information, including age, sex, thyroid function, antibody levels, and pathology results, were not available to the physicians during interpretation. This setting was designed to provide an image-only comparison with the model rather than to simulate the complete clinical diagnostic process.

### 2.3. Deep Learning Model Development

**Figure 2:**
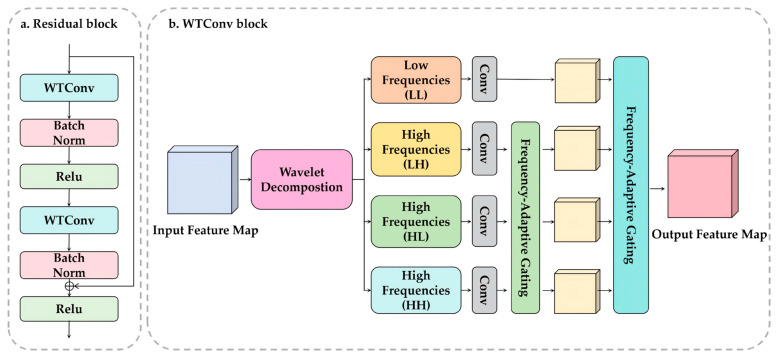
Architecture of the Frequency-Adaptive WT-ResNet. (**a**) Structure of the residual block. Each residual block consists of two WTConv layers, followed by batch normalization, with a ReLU activation after the first batch normalization and another ReLU activation after residual addition. The shortcut connection preserves the input features and facilitates gradient propagation. (**b**) Structure of the WTConv block. The input feature map is first decomposed by wavelet transform into one low-frequency subband (LL) and three high-frequency subbands (LH, HL, and HH). Each subband is processed by a convolutional operation. The high-frequency branches are further modulated by a frequency-adaptive gating mechanism to enhance discriminative fine-grained features and suppress less informative responses. The processed subbands are then fused to generate the output feature map.

A deep learning model named Frequency-Adaptive WT-ResNet was developed on the basis of a ResNet backbone for image-level classification of DLBCL, MALT lymphoma, and HT (Figure 2) [21,22]. The standard 3 × 3 convolution in each ResNet bottleneck block was replaced by a wavelet transform convolution (WTConv) module [23]. This design was intended to better preserve diagnostically relevant fine-grained sonographic details that may be attenuated during repeated convolution and pooling operations [24].

WTConv decomposes feature maps into one low-frequency component and three high-frequency subbands using a discrete wavelet transform. The low-frequency component captures coarse anatomical structures and lesion contours, whereas the high-frequency components capture finer textural and edge-related information. The model then performs convolution in the frequency domain and reconstructs the features, ensuring that critical microtextural cues are retained in deep layers and reducing misclassification caused by detail loss during downsampling.

To improve the representation of underrepresented classes, especially MALT lymphoma, a lightweight frequency-adaptive gating mechanism was introduced before inverse wavelet reconstruction. This module dynamically learns and adjusts the weights of different frequency channels based on feedback from the training process, thereby effectively improving feature separability between rare and common lesions in the frequency domain without relying on extensive data augmentation.

Gradient-weighted Class Activation Mapping (Grad-CAM) was applied to the final trained model to visualize image regions contributing to the model predictions.

### 2.4. Model Training

Input images were resized to 224 × 224 pixels. The wavelet transform used the Haar wavelet with one-level decomposition. WTConv replaced the 3 × 3 convolution in each ResNet bottleneck block. In the frequency-adaptive gating module, channel-wise weights were generated using global average pooling followed by a two-layer multilayer perceptron with a reduction ratio of 16 and a sigmoid activation function; the resulting weights were applied to the LH, HL, and HH high-frequency subbands before inverse wavelet reconstruction.

The network was trained using the Adam optimizer with an initial learning rate of 0.001, a batch size of 32, and 30 epochs. The loss function was cross-entropy. Training was performed on an NVIDIA RTX 4090 GPU with 24 GB memory.

### 2.5. Statistical Analysis

Statistical analyses were performed using Python 3.12 with the pandas, NumPy, and SciPy libraries. Categorical variables were presented as numbers and percentages. Intergroup comparisons of categorical variables were performed using the chi-squared test or Fisher’s exact test, as appropriate. A two-sided *p* value < 0.05 was considered statistically significant. The 95% confidence intervals (CIs) for diagnostic performance metrics were estimated using bootstrap resampling with 1000 iterations.

**Figure 3:**
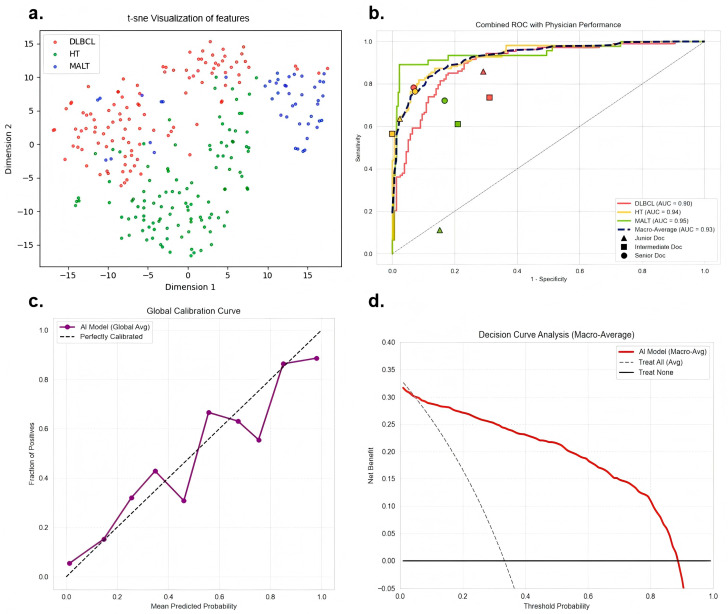
Performance of the deep learning model on the test set. (**a**) t-SNE visualization of feature distributions in the test set. (**b**) Receiver operating characteristic (ROC) curves for DLBCL, MALT lymphoma, and HT, together with the macro-average ROC curve. The operating points of the junior, intermediate, and senior physicians are overlaid for descriptive comparison. (**c**) Calibration curve of the model. The dashed diagonal line indicates perfect calibration. (**d**) Decision curve analysis (DCA) of the model based on macro-average performance.

For the comparison with physicians, model performance was summarized using ROC curves, whereas physicians produced single discrete classifications. The physician results were overlaid on the model ROC curves and compared descriptively rather than by formal paired AUC testing (Figure 3b). Inter-observer agreement analysis among the three physicians was performed. Since the diagnostic task involved three nominal categories, Fleiss’ kappa was used to evaluate the overall agreement, and pairwise Cohen’s kappa was used for comparisons between physicians.

The primary endpoint was image-level diagnostic performance for three-class classification of DLBCL, MALT lymphoma and HT. Secondary endpoints included class-specific performance for each disease category, comparison with ultrasound physicians, calibration analysis, decision curve analysis, and visual interpretability using Grad-CAM. Diagnostic performance was evaluated using the area under the receiver operating characteristic curve (AUC), balanced accuracy, sensitivity, specificity, positive predictive value (PPV), negative predictive value (NPV), and F1-score. For the exploratory external validation set, the same image-level metrics were calculated. ROC curves and confusion matrices were generated to visualize diagnostic performance. High-dimensional features extracted by the model were visualized using t-distributed stochastic neighbor embedding (t-SNE). Calibration curves and decision curve analysis (DCA) were used to assess prediction consistency and potential clinical utility.

## 3. Results

### 3.1. Cohort and Dataset Characteristics

A total of 290 patients with 313 lesions were included in this study, yielding 1294 static ultrasound images for analysis. The training set comprised 103 DLBCL lesions with 414 images, 51 MALT lymphoma lesions with 205 images, and 96 HT lesions with 410 images. The test set included 26 DLBCL lesions with 108 images, 13 MALT lymphoma lesions with 46 images, and 24 HT lesions with 111 images. All images from the same lesion were kept within the same data partition to avoid cross-partition leakage. The primary analysis was conducted at the image level.

In the DLBCL group, 88 patients (68.22%) were diagnosed by CNB and 41 patients (31.78%) by surgical biopsy. In the MALT lymphoma group, 34 patients (53.12%) were diagnosed by CNB and 30 patients (46.88%) by surgical biopsy. Both PTL subtypes were confirmed by histopathology with immunohistochemical analysis according to the WHO classification criteria. In the HT group, 9 patients (7.50%) underwent CNB, 80 patients (66.67%) underwent FNA, and 31 patients (25.83%) underwent surgical biopsy. FNA was mainly used to evaluate suspicious thyroid nodules or focal hypoechoic areas, with the aim of excluding malignancy and confirming Hashimoto-related lesions, rather than serving as the sole diagnostic method for clinically suspected PTL. Most HT patients underwent subsequent ultrasound follow-up at our institution, and no malignant progression was observed.

### 3.2. Clinical and Ultrasound Feature Analysis

The clinical and ultrasound characteristics of the three groups are summarized in Table 1. PTL is more common in patients aged 45 years and older, whereas HT is more frequent in younger women (*p* < 0.0001). For ultrasound features, abnormal cervical lymph nodes (*p* = 0.0367), lesion type (*p* = 0.0277) and morphology (*p* = 0.0079) showed significant differences between the DLBCL and MALT lymphoma groups. DLBCL more frequently presents as diffuse lesions with irregular morphology. Compared with HT, both DLBCL and MALT lymphoma showed a higher prevalence of abnormal cervical lymph nodes (*p* < 0.0001) and increased vascularity (MALT lymphoma vs. HT, *p* = 0.0007; DLBCL vs. HT, *p* = 0.0003).

### 3.3. Image-Level Diagnostic Performance of the Model

The proposed Frequency-Adaptive WT-ResNet showed favorable performance for differentiation of DLBCL, MALT lymphoma and HT. The t-SNE plot demonstrated separation of the three categories in feature space, suggesting that the model learned discriminative image representations (Figure 3a). In the test set, the model achieved a macro-average AUC value of 0.927 (95% CI: 0.889–0.960), with the highest performance observed for MALT lymphoma (AUC = 0.946, 95% CI: 0.900–0.981), followed by HT (AUC = 0.937, 95% CI: 0.908–0.963) and DLBCL (AUC = 0.899, 95% CI: 0.858–0.935). The macro-average balanced accuracy was 0.866 (95% CI: 0.820–0.910), and the macro-average F1-score was 0.823 (95% CI: 0.753–0.882) (Table 2). The calibration curve showed good agreement between predicted and observed probabilities (Figure 3c). Decision curve analysis further suggested that compared with the full-treatment or non-treatment strategy within a wide threshold probability range, the model provided significantly higher net benefits, highlighting its potential clinical application value (Figure 3d).

### 3.4. Physician Performance

Among the three readers, the senior physician achieved the best overall performance, with a macro-average balanced accuracy of 0.827 (95% CI: 0.759–0.892) (Table 3). The intermediate physician achieved a macro-average balanced accuracy of 0.732 (95% CI: 0.656–0.809), which was higher than that of the junior physician. Physician performance appeared to vary with experience level. Across readers, the balanced accuracy for MALT lymphoma remained relatively low, whereas the specificity for HT was consistently high, suggesting that HT was relatively easier to identify than lymphoma on static ultrasound images.

Inter-observer agreement among the three physicians was further assessed using kappa statistics. The overall Fleiss’s kappa was 0.443, indicating moderate agreement among the three readers. Pairwise Cohen’s kappa values ranged from 0.328 to 0.548 (Table 4). Specifically, the agreement was moderate between the senior and intermediate physicians (κ = 0.458; 41/63 concordant cases, 65.1%) and between the senior and junior physicians (κ = 0.548; 45/63 concordant cases, 71.4%), whereas the agreement between the intermediate and junior physicians was fair (κ = 0.328; 38/63 concordant cases, 60.3%). Complete agreement among all three physicians was observed in 49.2% of cases. Notably, complete agreement was lowest for MALT lymphoma, suggesting that MALT lymphoma was more challenging to differentiate sonographically from DLBCL and HT.

When physicians’ operating points were compared with the model ROC curves, most reader points were located below or within the ROC curves, indicating lower sensitivity at comparable specificity levels (Figure 3b). For MALT lymphoma, the reader points were farther from the upper-left corner than the model ROC curve, suggesting that the model may have provided better discrimination for this underrepresented subtype in the static-image setting.

### 3.5. Grad-CAM Visualization

**Figure 4:**
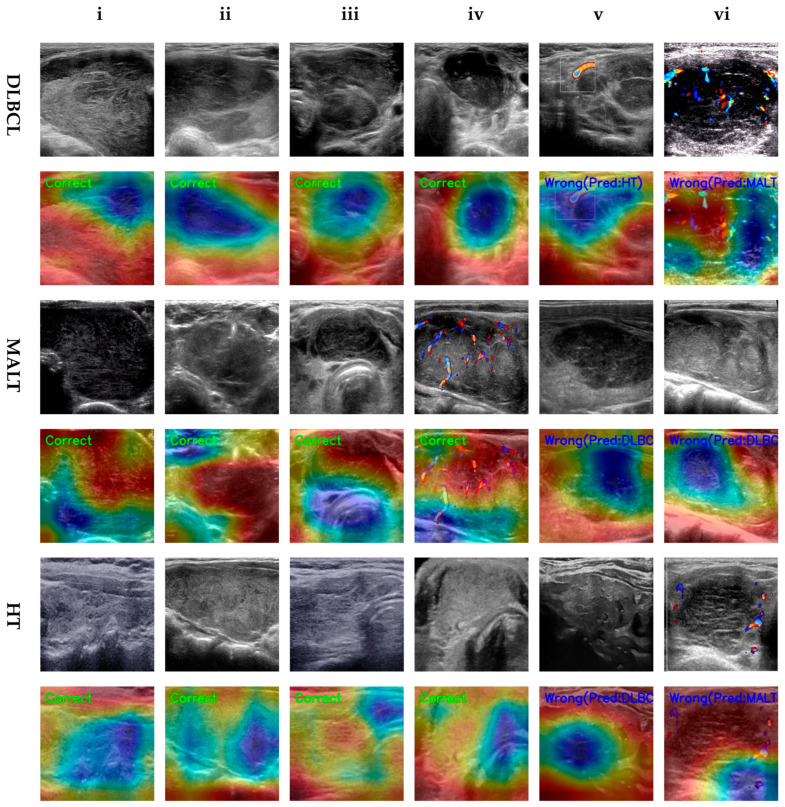
Representative ultrasound images and Grad-CAM heatmaps of DLBCL, HT, and MALT lymphoma. Red regions indicate higher contribution to model prediction, whereas blue regions indicate lower contribution. Columns (**i**)–(**vi**) show representative cases. The upper rows display the ultrasound images, and the lower rows show the corresponding Grad-CAM heatmaps. The HT group was characterized by diffuse parenchymal involvement, with or without focal inflammatory hypoechoic areas (also referred to as Hashimoto’s nodules), rather than by isolated tumor-like lesions used for lesion feature analysis.

Grad-CAM was used to visualize image regions contributing to model predictions (Figure 4). It provides only auxiliary visual evidence and should not be interpreted as a direct causal explanation of the model’s decision-making process.

In DLBCL, model activation was mainly concentrated within the lesion itself, with gradually increasing intensity from the center toward the periphery, covering the lesion margin and heterogeneous echotextural features of the surrounding tissue. This pattern is consistent with the typical ultrasound manifestations of DLBCL, including a strong sense of local architectural disruption, heterogeneous internal echogenicity and ill-defined margins. In MALT lymphoma, the activation areas extended from the lesion center to the adjacent peripheral region, with the central activation mainly corresponding to focal areas of heterogeneous hypoechogenicity. MALT lymphoma usually shows relatively indolent growth, and some cases may present as focal hypoechoic areas with relatively clear boundaries from the surrounding normal glandular tissue. Therefore, the model may rely on the internal hypoechoic components and the interface with adjacent normal tissue for classification. The activation areas were more diffusely distributed and covered a large proportion of the thyroid parenchyma in HT. This finding is consistent with the typical sonographic features of HT, including disorganized glandular architecture and diffuse parenchymal echotextural heterogeneity. The model’s recognition of HT may depend more on the overall thyroid background rather than on a single isolated tumor-like lesion.

Although the model demonstrated good overall performance, it failed to fully capture subtype-specific internal microtextures, global lesion morphology, and diffuse background information in some challenging cases. For example, in DLBCL cases that the model misclassified as MALT lymphoma, the lesions were relatively localized, showed relatively clear demarcation from the surrounding tissue and lacked typical highly aggressive internal features. These features therefore led the model to assign the cases to MALT lymphoma. In HT cases misclassified as DLBCL or MALT lymphoma, focal inflammatory hypoechoic areas or Hashimoto nodules produced localized tumor-like appearances, and the corresponding activation patterns also resembled those of PTL.

### 3.6. Exploratory External Validation

**Figure 5:**
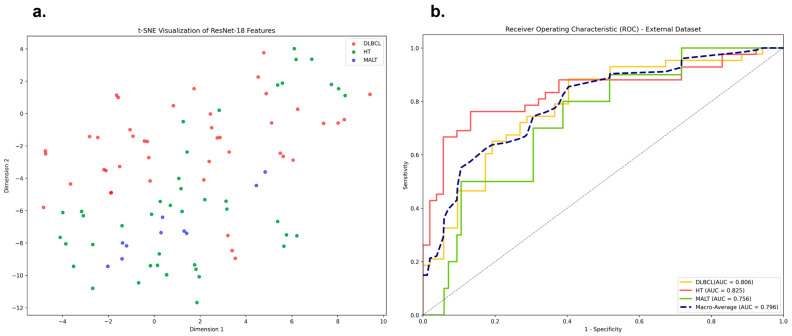
Performance of the deep learning model on exploratory external validation set. (**a**) t-SNE visualization of feature distributions in the exploratory external validation cohort. (**b**) ROC curves of the model in the exploratory external validation cohort.

In this independent institutional dataset, the model achieved a macro-average AUC of 0.796 (95% CI: 0.686–0.888). The class-specific AUCs were 0.806 (95% CI: 0.711–0.891) for DLBCL, 0.825 (95% CI: 0.727–0.909) for HT, and 0.756 (95% CI: 0.620–0.865) for MALT lymphoma (Table 5). The t-SNE visualization showed partial separation among the three diagnostic categories, but substantial overlap remained between DLBCL and HT (Figure 5a). The ROC curves further indicated that the model retained a certain degree of discriminative ability in the external cohort (Figure 5b). This reduction may be attributable to the very small and imbalanced external sample, especially the limited number of MALT lymphoma lesions, as well as potential domain shift related to differences in ultrasound equipment, imaging protocols, operator technique, and image quality across institutions. Therefore, the external validation results should be interpreted as preliminary and underpowered, and larger multicenter cohorts remain necessary to confirm model generalizability.

## 4. Discussion

In this study, we developed and evaluated a frequency-adaptive deep learning model based on BMUS and CDUS images for the image-level differentiation of DLBCL, MALT lymphoma, and HT. The model achieved favorable diagnostic performance in the test set and showed higher overall performance than three ultrasound physicians in a static-image comparison. These findings suggest that a frequency-adaptive deep learning approach may provide useful decision support for the preoperative assessment of thyroid lesions with overlapping sonographic appearances.

The exploratory external validation further showed that the model retained some discriminative ability in an independent institutional dataset. It highlights the potential transferability of the image-level model. The relatively lower performance in this exploratory external dataset may be attributable to the very small and imbalanced external sample, especially the limited number of MALT lymphoma lesions. The external images were obtained from a different institution, which may have introduced domain shift related to differences in ultrasound equipment, imaging protocols, operator technique and image quality. Therefore, the external validation results should be interpreted cautiously.

In our static ultrasound image classification task, inter-observer agreement among ultrasound physicians was only fair to moderate. This finding indicates limited reproducibility of physician interpretation when differentiating DLBCL, MALT lymphoma, and HT based solely on single static ultrasound images. In particular, the relatively low agreement for MALT lymphoma further highlights the diagnostic difficulty caused by overlapping sonographic features and supports the potential value of AI-assisted image interpretation for providing more consistent image-level diagnostic support.

The HT cohort was not selected from the general population of clinically diagnosed patients. Only pathology-confirmed HT cases were included. These patients underwent pathological examination because of suspicious sonographic findings, including suspicious thyroid nodules or focal hypoechoic areas, indistinct margins, irregular morphology, punctate echogenic foci or microcalcifications, a taller-than-wide shape, suspected capsular invasion, and suspicious cervical lymph nodes. The main indications for biopsy or surgery were to establish a definitive diagnosis. After excluding HT patients with concurrent thyroid carcinoma in the ipsilateral thyroid lobe, we further aimed to determine whether these patients had HT alone or HT associated with PTL. HT cohort confirmed by pathology provided a clinically relevant control group for evaluating PTL-like sonographic presentations.

The favorable performance of the proposed model may be related to its ability to preserve diagnostically relevant fine-grained sonographic information. In conventional convolutional networks, repeated convolution and pooling operations may attenuate subtle high-frequency image features. In our framework, WTConv was introduced to decompose feature maps into low- and high-frequency components, while the frequency-adaptive gating module was designed to modulate high-frequency responses. By effectively retaining critical high-frequency components, such as internal texture roughness and edge sharpness, the model may better decode the highly overlapping ultrasonic phenotype features of DLBCL, MALT lymphoma, and HT. In addition, Grad-CAM visualizations also suggested class-dependent attention patterns. DLBCL showed activation mainly concentrated within the lesion body and gradually increasing from the center toward the periphery; MALT lymphoma showed activation extending from the lesion center to the surrounding region and mainly concentrated in locally heterogeneous hypoechoic areas; and HT showed more diffuse activation covering a relatively large extent of the parenchyma.

The clinical relevance of this task lies in the substantial overlap between PTL subtypes and HT on ultrasound. PTL often arises in the background of HT [25], and both entities may present with diffuse enlargement, marked hypoechogenicity, and heterogeneous echotexture. In addition, PTL subtypes distinction is clinically meaningful because DLBCL and MALT lymphoma differ in biological behavior and treatment strategy. DLBCL is more aggressive and is characterized by diffuse infiltration of large atypical lymphocytes, resulting in relatively heterogeneous hypoechogenicity, ill-defined boundary and posterior acoustic enhancement on ultrasound [26,27]. MALT lymphoma demonstrates a more indolent and localized growth pattern, with lymphoid follicle formation, lymphoepithelial lesions and prominent plasma cell infiltration, and typically presents as predominantly solid hypoechoic nodules with linear or reticular hyperechoic components and posterior acoustic enhancement [28]. Accurate identification of PTL and HT may help avoid misdiagnosing malignant lymphoma as benign inflammatory disease and thereby reduce delays in biopsy and pathological work-up. Precise differentiation of DLBCL from MALT lymphoma is also clinically important, as it may prevent aggressive tumors from being mistaken for indolent lesions and thus avoid delays in appropriate management.

Previous deep learning studies based on thyroid ultrasound have mainly focused on grayscale imaging. In contrast, vascular information may provide complementary diagnostic value beyond morphology alone. Previous contrast-enhanced ultrasound studies have shown significant differences in vascular distribution patterns between PTL and HT lesions, with PTL often exhibiting mixed vascularity and HT lesions more commonly showing peripheral vascular flow [29]. No significant statistical difference in Adler blood flow grade was found between the DLBCL and MALT lymphoma groups in this study. However, when the two PTL subtypes were considered together, a significant difference was observed between PTL and HT. PTL mainly exhibited grade 2 blood flow signals, while HT was mainly characterized by grade 1 blood flow signals. Moreover, deep learning models using ultrasound cine loops have shown better performance than static 2D images, as static ultrasound images alone may not fully reflect the heterogeneity of lesion characteristics [30].

Several AI-assisted thyroid ultrasound tools have been developed and some have entered clinical use, including systems such as S-Detect for Thyroid (Samsung Medison), AmCAD-UT (AmCad BioMed), Koios DS (Koios Medical), Medo Thyroid (Medo AI) and other TI-RADS-oriented CAD platforms [31,32,33,34]. However, these tools and most published thyroid ultrasound AI studies are designed primarily for thyroid nodule detection, feature extraction, TI-RADS-based risk stratification, or benign-malignant classification of nodules [35]. They are not specifically designed to identify PTL, or to differentiate DLBCL from MALT lymphoma, or to distinguish PTL from HT with overlapping diffuse low-echoic or inflammatory parenchymal changes. Therefore, direct performance comparison with these products was not feasible in this retrospective cohort, because their output categories do not correspond to the three-class diagnostic task in the present study. The novelty of our work lies in using BMUS and CDUS images for image-level multi-class differentiation of DLBCL, MALT lymphoma and HT, which addresses a clinically specific but underexplored diagnostic problem. This framework may potentially be adapted to other imaging tasks and modalities, including CT, MRI, and PET.

The present study has several limitations. First, its retrospective design from a single center and the use of different ultrasound machines over a long time span may have introduced selection bias. In addition, domain shift caused by differences in equipment manufacturers, image quality and operator experience may have further affected the robustness and generalizability of the model. Standardized preprocessing was applied to reduce acquisition-dependent intensity variation, but the number of cases per vendor and per acquisition period was insufficient for reliable stratified performance analysis. Although an exploratory external validation cohort was obtained from Sun Yat-sen Memorial Hospital, the small sample size and marked class imbalance limited statistical reliability. Future studies should continue to establish larger prospective multicenter cohorts with standardized image acquisition and predefined subgroup analyses by device, institution, and time period to evaluate model robustness. Second, the HT cohort was pathology-based rather than population-based. This design was intended to construct a clinically relevant control group for PTL differentiation, but it may also introduce selection bias. Future studies should include both pathology-confirmed HT cases and clinically diagnosed HT cases to evaluate model generalizability. Third, as chemotherapy and radiotherapy are the main treatments for PTL, surgery is generally reserved for relieving cervical compression, resulting in limited surgically resected specimens. In addition, FNA was mainly used to evaluate suspicious thyroid nodules or focal hypoechoic areas to exclude malignancy and confirm Hashimoto-related lesions, but its diagnostic ability to distinguish HT from PTL remains limited. Fourth, the distribution of the three diagnostic categories was imbalanced in this study. Although a frequency-adaptive gating mechanism was incorporated to mitigate the influence of class imbalance, residual bias may still have affected model training and performance estimation. Fifth, static images were analyzed instead of cine clips, so future investigations should incorporate ultrasound cine clips and additional ultrasound modalities, such as ultrasound elastography and contrast-enhanced ultrasound to capture more comprehensive lesion characteristics.

## 5. Conclusions

This study demonstrates that a frequency-adaptive WT-ResNet deep learning model based on BMUS and CDUS images achieves promising image-level performance for differentiating DLBCL, MALT lymphoma, and HT. By integrating wavelet transform and a frequency-adaptive gating mechanism, the model was specifically optimized and outperformed three ultrasound physicians in a blinded static-image comparison. Exploratory external validation in a small independent cohort suggested partial external discriminative ability.

The proposed deep learning model may serve as an image-level decision-support tool rather than an independent lesion- or patient-level diagnostic system. In clinical practice, its output should be integrated with multi-view ultrasound assessment, clinical information, and pathological findings to support preoperative evaluation, reduce potential diagnostic delays, and guide appropriate biopsy strategies. Further multicenter, external, and prospective validation is required before clinical implementation.

## Figures and Tables

**Table 1:** Comparison of clinic and ultrasound features between groups of MALT, DLBCL lymphoma and HT.

Groups/ Indicators	MALT (64)	DLBCL (129)	HT (120)	χ^2^	*p*-Value	*p*-Value (MALT vs. DLBCL)	*p*-Value (MALT vs. HT)	*p*-Value (DLBCL vs. HT)
Site				3.1174	0.5384	0.4794	0.4348	0.4329
Right lobe	29 (45.31%)	69 (53.49%)	65 (54.17%)					
Left lobe	33 (51.56%)	58 (44.96%)	50 (41.67%)					
Isthmus	2 (3.12%)	2 (1.55%)	5 (4.17%)					
Sex				15.2505	0.0005	0.8843	0.0008	0.0003
Man	18 (28.12%)	35 (27.13%)	11 (9.17%)					
Woman	46 (71.88%)	94 (72.87%)	109 (90.83%)					
Age				74.0798	<0.0001	0.0839	<0.0001	<0.0001
<45	10 (15.62%)	10 (7.75%)	51 (42.50%)					
45–60	23 (35.94%)	37 (28.68%)	51 (42.50%)					
>60	31 (48.44%)	82 (63.57%)	18 (15.00%)					
Specimen				8.4769	0.0144	0.0407	0.0039	0.3008
Biopsy	34 (53.12%)	88 (68.22%)	89 (74.17%)					
Resection	30 (46.88%)	41 (31.78%)	31 (25.83%)					
HT background				12.2795	0.0022	0.6105	0.0046	0.0004
Present	59 (92.19%)	116 (89.92%)	120 (100.00%)					
Absent	5 (7.81%)	13 (10.08%)	0 (0.00%)					
Lesion Type				7.1692	0.0277	0.0277	-	-
Diffuse	30 (46.88%)	78 (60.47%)	-					
Nodular	30 (46.88%)	36 (27.91%)	-					
Mixed	4 (6.25%)	15 (11.63%)	-					
Abnormal CLN				59.8963	<0.0001	0.0367	<0.0001	<0.0001
Present	26 (40.62%)	73 (56.59%)	12 (10.00%)					
Absent	38 (59.38%)	56 (43.41%)	108 (90.00%)					
Adler grade				21.1393	0.0003	0.5742	0.0007	0.0003
Grade 1	21 (32.81%)	51 (39.53%)	68 (56.67%)					
Grade 2	33 (51.56%)	63 (48.84%)	29 (24.17%)					
Grade 3	10 (15.62%)	15 (11.63%)	23 (19.17%)					
Boundary				3.1036	0.0781	0.0781	-	-
Distinct	27 (42.19%)	38 (29.46%)	-					
Indistinct	37 (57.81%)	91 (70.54%)	-					
Morphology				8.2380	0.0041	0.0079	-	-
Regular	10 (15.62%)	5 (3.88%)	-					
Irregular	54 (84.38%)	124 (96.12%)	-					
Aspect Ratio				1.5119	0.2189	0.5521	-	-
Wider-than-tall	64 (100.00%)	126 (97.67%)	-					
Taller-than-wide	0 (0.00%)	3 (2.33%)	-					
Echogenicity				27.3216	<0.0001	0.0973	0.0010	<0.0001
Hypo echo	56 (87.50%)	100 (77.52%)	119 (99.17%)					
Marked hypo echo	8 (12.50%)	29 (22.48%)	1 (0.83%)					

Note: Overall *p*-values were calculated using Pearson’s chi-square test. For pairwise comparisons, Pearson’s chi-square test was used for multi-category variables; for 2 × 2 tables, continuity-corrected chi-square was used when all observed cell counts were ≥5, otherwise Fisher’s exact test was applied. For lesion type, boundary, morphology, and aspect ratio, HT was not assessed because the HT control group was defined by diffuse thyroid parenchymal involvement or focal inflammatory hypoechoic areas rather than isolated tumor lesions with measurable margins. Therefore, the HT column was marked as “-”, and the corresponding statistical analyses were performed using only the MALT lymphoma and DLBCL groups.

**Table 2:** Diagnostic performance of the deep learning model in the test set.

Groups	AUC (95% CI)	Balanced Acc (95% CI)	Sensitivity (95% CI)	Specificity (95% CI)	PPV (95% CI)	NPV (95% CI)	F1-Score (95% CI)
DLBCL	0.899 (0.858–0.935)	0.830 (0.785–0.876)	0.852 (0.778–0.918)	0.809 (0.747–0.869)	0.754 (0.679–0.825)	0.888 (0.832–0.939)	0.800 (0.737–0.853)
HT	0.937 (0.908–0.963)	0.841 (0.797–0.887)	0.748 (0.664–0.826)	0.935 (0.895–0.969)	0.892 (0.826–0.950)	0.837 (0.785–0.892)	0.814 (0.751–0.870)
MALT lymphoma	0.946 (0.900–0.981)	0.925 (0.877–0.967)	0.891 (0.792–0.974)	0.959 (0.930–0.982)	0.820 (0.711–0.923)	0.977 (0.954–0.995)	0.854 (0.769–0.922)
Macro-average	0.927 (0.889–0.960)	0.866 (0.820–0.910)	0.830 (0.744–0.906)	0.901 (0.857–0.940)	0.822 (0.739–0.900)	0.901 (0.857–0.942)	0.823 (0.753–0.882)

**Table 3:** Diagnostic performance of physicians.

Physicians	Groups	Balanced Acc (95% CI)	Sensitivity (95% CI)	Specificity (95% CI)	PPV (95% CI)	NPV (95% CI)	F1-Score (95% CI)
Junior	DLBCL	0.784 (0.732–0.840)	0.858 (0.784–0.919)	0.709 (0.619–0.792)	0.752 (0.670–0.825)	0.830 (0.742–0.900)	0.802 (0.746–0.858)
	HT	0.806 (0.753–0.862)	0.635 (0.537–0.742)	0.976 (0.945–1.000)	0.947 (0.885–1.000)	0.796 (0.727–0.860)	0.761 (0.680–0.838)
	MALT	0.480 (0.409–0.567)	0.111 (0.000–0.278)	0.848 (0.789–0.897)	0.065 (0.000–0.160)	0.910 (0.868–0.950)	0.082 (0.000–0.194)
	Macro-average	0.690 (0.631–0.756)	0.535 (0.441–0.646)	0.844 (0.784–0.896)	0.588 (0.518–0.662)	0.845 (0.779–0.903)	0.548 (0.475–0.630)
Intermediate	DLBCL	0.713 (0.653–0.774)	0.736 (0.651–0.821)	0.689 (0.602–0.778)	0.709 (0.625–0.789)	0.717 (0.625–0.802)	0.722 (0.654–0.785)
	HT	0.782 (0.729–0.835)	0.565 (0.458–0.670)	1.000 (1.000–1.000)	1.000 (1.000–1.000)	0.770 (0.700–0.831)	0.722 (0.629–0.803)
	MALT	0.701 (0.587–0.816)	0.611 (0.389–0.833)	0.791 (0.730–0.848)	0.216 (0.098–0.321)	0.956 (0.924–0.987)	0.319 (0.161–0.447)
	Macro-average	0.732 (0.656–0.809)	0.637 (0.499–0.775)	0.827 (0.777–0.875)	0.642 (0.574–0.703)	0.814 (0.750–0.873)	0.588 (0.481–0.678)
Senior	DLBCL	0.858 (0.812–0.902)	0.783 (0.700–0.862)	0.932 (0.881–0.978)	0.922 (0.863–0.973)	0.807 (0.736–0.874)	0.847 (0.794–0.898)
	HT	0.846 (0.793–0.898)	0.765 (0.677–0.854)	0.927 (0.875–0.971)	0.878 (0.797–0.950)	0.852 (0.786–0.912)	0.818 (0.746–0.880)
	MALT	0.777 (0.670–0.877)	0.722 (0.500–0.917)	0.832 (0.781–0.882)	0.289 (0.163–0.419)	0.970 (0.941–0.994)	0.413 (0.254–0.543)
	Macro-average	0.827 (0.759–0.892)	0.757 (0.626–0.877)	0.897 (0.845–0.944)	0.696 (0.608–0.781)	0.876 (0.821–0.927)	0.692 (0.598–0.774)

**Table 4:** Pairwise inter-observer agreement among ultrasound physicians.

Physicians	Concordant Cases/Total	Simple Concordance Rate	Cohen’s κ	Interpretation of Concordance
Senior vs. Intermediate	41/63	65.1%	0.458	Moderate
Senior vs. Junior	45/63	71.4%	0.548	Moderate
Intermediate vs. Junior	38/63	60.3%	0.328	Fair

**Table 5:** Diagnostic performance of the deep learning model in the exploratory external validation cohort.

Groups	AUC (95% CI)	Balanced Acc (95% CI)	Sensitivity (95% CI)	Specificity (95% CI)	PPV (95% CI)	NPV (95% CI)	F1-Score (95% CI)
DLBCL	0.806 (0.711–0.891)	0.675 (0.548–0.799)	0.581 (0.444–0.725)	0.769 (0.651–0.873)	0.676 (0.518–0.821)	0.690 (0.577–0.800)	0.625 (0.500–0.738)
HT	0.825 (0.727–0.909)	0.565 (0.483–0.647)	0.167 (0.065–0.294)	0.962 (0.900–1.000)	0.778 (0.500–1.000)	0.593 (0.494–0.695)	0.275 (0.118–0.440)
MALT lymphoma	0.756 (0.620–0.865)	0.659 (0.457–0.813)	0.800 (0.500–1.000)	0.518 (0.414–0.625)	0.163 (0.068–0.281)	0.957 (0.881–1.000)	0.271 (0.122–0.426)
Macro-average	0.796 (0.686–0.888)	0.633 (0.496–0.753)	0.516 (0.337–0.673)	0.750 (0.655–0.833)	0.539 (0.362–0.700)	0.746 (0.651–0.832)	0.390 (0.247–0.535)

## Data Availability

The original contributions presented in this study are included in the article. Further inquiries can be directed to the corresponding author.

## Supplementary Materials

The following supporting information can be downloaded at https://www.mdpi.com/article/10.3390/diagnostics16121909/s1, TRIPOD+AI checklist.

## Abbreviations

The following abbreviations are used in this manuscript:

PTL	Primary Thyroid Lymphoma
DLBCL	Diffuse Large B-cell Lymphoma
MALT	Mucosa-Associated Lymphoid Tissue
HT	Hashimoto’s Thyroiditis
BMUS	B-mode Ultrasound
CDUS	Color Doppler Ultrasound
WTConv	Wavelet Transform Convolution
ResNet	Residual Network
AUC	Area under the receiver operating characteristic curve
ROC	Receiver Operating Characteristic
Grad-CAM	Gradient-weighted Class Activation Mapping
FNA	Fine-Needle Aspiration
CNB	Core-Needle Biopsy
H&E	Hematoxylin and Eosin
^18^F-FDG PET	^18^F-Fluorodeoxyglucose Positron Emission Tomography
PPV	Positive Predictive Value
NPV	Negative Predictive Value
Acc	Accuracy
CI	Confidence Interval
t-SNE	t-distributed Stochastic Neighbor Embedding
DCA	Decision Curve Analysis
CT	Computed Tomography
MRI	Magnetic Resonance Imaging
