# Supplementary material for: Deep Learning Based on B-Mode and Color Doppler Ultrasound for Differentiation of Primary Thyroid Lymphoma and Hashimoto’s Thyroiditis: A Retrospective Single-Center Study
Source: Diagnostics (Basel). 2026 Jun 19;16(12):1909. doi: 10.3390/diagnostics16121909 (PMC13298520; doi:10.3390/diagnostics16121909)
Supplement: Supplementary file 1 [file diagnostics-16-01909-s001.zip › diagnostics-4386152-supplementary.pdf]

TRIPOD+AI

Section/Topic	Item	Development / evaluation	Checklist item	Reported on page
**TITLE**				
Title	1	D;E	Identify the study as developing or evaluating the performance of a multivariable prediction model, the target population, and the outcome to be predicted	Title page
**ABSTRACT**				
Abstract	2	D;E	See TRIPOD+AI for Abstracts checklist	Page 1, Abstract
**INTRODUCTION**				
Background	3a	D;E	Explain the healthcare context (including whether diagnostic or prognostic) and rationale for developing or evaluating the prediction model, including references to existing models	Page 2, Introduction, paras. 1-3
	3b	D;E	Describe the target population and the intended purpose of the prediction model in the context of the care pathway, including its intended users (e.g., healthcare professionals, patients, public)	Page 2, Introduction, para. 4<br>Page 15, Conclusions
	3c	D;E	Describe any known health inequalities between ociodemographic groups	Page 8, Table 1
Objectives	4	D;E	Specify the study objectives, including whether the study describes the development or validation of a prediction model (or both)	Page 1, Abstract<br>Page 2, Introduction, para. 4
**METHODS**				
Data	5a	D;E	Describe the sources of data separately for the development and evaluation datasets (e.g., randomized trial, cohort, routine care or registry data), the rationale for using these data, and representativeness of the data	Page 3, Materials and Methods, 2.1 Study Design and Population (internal development/test cohort and exploratory external validation cohort)<br>Page 3, Figure 1
	5b	D;E	Specify the dates of the collected participant data, including start and end of participant accrual; and, if applicable, end of follow-up	Page 3, Materials and Methods, 2.1 (internal cohort: September 2002 to September 2024; external validation cohort: between December 2015 and December 2025)
Participants	6a	D;E	Specify key elements of the study setting (e.g., primary care, secondary care, general population) including the number and location of centres	Page 3, Materials and Methods, 2.1 (internal cohort from West China Hospital, Sichuan University, Chengdu, China; exploratory external validation from Sun Yat-sen Memorial Hospital, Sun Yat-sen University, Guangzhou, China)
	6b	D;E	Describe the eligibility criteria for study participants	Page 3, Materials and Methods, 2.1 (inclusion and exclusion criteria)
	6c	D;E	Give details of any treatments received, and how they were handled during model development or evaluation, if relevant	Not applicable
Data preparation	7	D;E	Describe any data pre-processing and quality checking, including whether this was similar across relevant sociodemographic groups	Page 4, Materials and Methods, 2.2 Images Acquisition (same preprocessing pipeline applied to training, test, and validation images)
Outcome	8a	D;E	Clearly define the outcome that is being predicted and the time horizon, including how and when assessed, the rationale for choosing this outcome, and whether the method of outcome assessment is consistent across sociodemographic groups	Page 1, Abstract<br>Page 6, Methods; 2.5 Statistical Analysis (primary endpoint and three-class image-level outcome)
	8b	D;E	If outcome assessment requires subjective interpretation, describe the qualifications and demographic characteristics of the outcome assessors	Page 3, Materials and Methods, 2.1 (pathological confirmation by CNB/surgery/FNA as applicable)
	8c	D;E	Report any actions to blind assessment of the outcome to be predicted	Page 4, Materials and Methods, 2.2 (reviewers blinded to clinical/pathological results); reader comparison also blinded
Predictors	9a	D	Describe the choice of initial predictors (e.g., literature, previous models, all available predictors) and any pre-selection of predictors before model building	Page 4-6, Materials and Methods, 2.3-2.4 (BMUS and CDUS images)
	9b	D;E	Clearly define all predictors, including how and when they were measured (and any actions to blind assessment of predictors for the outcome and other predictors)	Page 4-6, Materials and Methods, 2.2 and 2.4 (BMUS/CDUS acquisition, image preprocessing, resizing / normalization)
	9c	D;E	If predictor measurement requires subjective interpretation, describe the qualifications and demographic characteristics of the predictor assessors	Page 4, Materials and Methods, 2.2 (two physicians >=5 years; senior radiologist 20 years resolved discrepancies)
Sample size	10	D;E	Explain how the study size was arrived at (separately for development and evaluation), and justify that the study size was sufficient to answer the research question. Include details of any sample size calculation	Page 3/7, Materials and Methods, 2.1 and 3.1 (available consecutive cohort)
Missing data	11	D;E	Describe how missing data were handled. Provide reasons for omitting any data	Page 3, Materials and Methods, 2.1 (incomplete clinical/ultrasound data and poor-quality images excluded)
Analytical methods	12a	D	Describe how the data were used (e.g., for development and evaluation of model performance) in the analysis, including whether the data were partitioned, considering any sample size requirements	Page 3/6, Materials and Methods, 2.1 and 2.5 (lesion-level 8:2 split for internal development/test; independent exploratory external validation; image-level primary analysis)

TRIPOD+AI

Section/Topic	Item	Development / evaluation	Checklist item	Reported on page
**TITLE**				
	12b	D	Depending on the type of model, describe how predictors were handled in the analyses (functional form, rescaling, transformation, or any standardization).	Page 4-6, Materials and Methods, 2.2 and 2.4 (resizing, per-image standardization/normalization)
	12c	D	Specify the type of model, rationale, all model-building steps, including any hyperparameter tuning, and method for internal validation	Page 4-6, Materials and Methods, 2.3-2.4 (Frequency-Adaptive WT-ResNet, WTConv, gating module, Adam, learning rate, batch size, epochs)
	12d	D;E	Describe if and how any heterogeneity in estimates of model parameter values and model performance was handled and quantified across clusters (e.g., hospitals, countries). See TRIPOD-Cluster for additional considerations[3]	Page 4, Exploratory external validation performed in an independent institution; no formal cluster-stratified heterogeneity analysis. Page 14-15, Limitations discuss device, institution, time-period heterogeneity and domain shift
	12e	D;E	Specify all measures and plots used (and their rationale) to evaluate model performance (e.g., discrimination, calibration, clinical utility) and, if relevant, to compare multiple models	Page 6, Materials and Methods, 2.5 (AUC, balanced accuracy, sensitivity, specificity, PPV, NPV, F1, ROC, confusion matrix, calibration, DCA, t-SNE, external validation metrics, and inter-observer agreement using Fleiss/Cohen kappa)
	12f	E	Describe any model updating (e.g., recalibration) arising from the model evaluation, either overall or for particular sociodemographic groups or settings	Not applicable
	12g	E	For model evaluation, describe how the model predictions were calculated (e.g., formula, code, object, application programming interface)	Page 4-6, Materials and Methods, 2.3-2.5; predictions generated by the trained Frequency-Adaptive WT-ResNet and evaluated on internal test and exploratory external validation images
Class imbalance	13	D;E	If class imbalance methods were used, state why and how this was done, and any subsequent methods to recalibrate the model or the model predictions	Page 4-5, Materials and Methods, 2.3; Page 14-15, Discussion/Limitations (frequency-adaptive gating to mitigate class imbalance; residual bias discussed)
Fairness	14	D;E	Describe any approaches that were used to address model fairness and their rationale	Page 8, Demographic differences reported in Table 1; Page 15, Limitations discussed external validation/domain-shift and generalizability
Model output	15	D	Specify the output of the prediction model (e.g., probabilities, classification). Provide details and rationale for any classification and how the thresholds were identified	Page 6, Materials and Methods, 2.5; Abstract/Results (three-class image-level classification with probabilistic performance evaluation)
Training versus evaluation	16	D;E	Identify any differences between the development and evaluation data in healthcare setting, eligibility criteria, outcome, and predictors	Page 3, Materials and Methods, 2.1; Page 7/12, Results 3.1/3.6 (same internal setting/criteria/outcome/predictors for training/test split; exploratory external validation from a different institution with limited and imbalanced sample)
Ethical approval	17	D;E	Name the institutional research board or ethics committee that approved the study and describe the participant-informed consent or the ethics committee waiver of informed consent	Page 3, Materials and Methods, 2.1; Institutional Review Board Statement (West China Hospital, Sichuan University, No. 2026292; consent waived)
**OPEN SCIENCE**				
Funding	18a	D;E	Give the source of funding and the role of the funders for the present study	Page 15, Funding statement (Key Research and Development Programme of Sichuan Province, 2024YFFK0226)
Conflicts of interest	18b	D;E	Declare any conflicts of interest and financial disclosures for all authors	Page 16, Conflicts of Interest statement
Protocol	18c	D;E	Indicate where the study protocol can be accessed or state that a protocol was not prepared	Not applicable
Registration	18d	D;E	Provide registration information for the study, including register name and registration number, or state that the study was not registered	Not applicable
Data sharing	18e	D;E	Provide details of the availability of the study data	Page 16, Data Availability Statement
Code sharing	18f	D;E	Provide details of the availability of the analytical code	Not applicable
**PATIENT and PUBLIC INVOLVEMENT**				
Patient and Public Involvement	19	D;E	Provide details of any patient and public involvement during the design, conduct, reporting, interpretation, or dissemination of the study or state no involvement.	Not applicable
**RESULTS**				
Participants	20a	D;E	Describe the flow of participants through the study, including the number of participants with and without the outcome and, if applicable, a summary of the follow-up time. A diagram may be helpful.	Page 7/ 12, Results, 3.1 and 3.6; Page3, Figure 1 (internal cohort flow and external validation cohort description)
	20b	D;E	Report the characteristics overall and, where applicable, for each data source or setting, including the key dates, key predictors (including demographics), treatments received, sample size, number of outcome events, follow-up time, and amount of missing data. A table may be helpful. Report any differences across key demographic groups.	Page 7/ 12, Results, 3.1-3.2 and 3.6; Page 8/ 12, Tables 1 and 5 (internal cohort characteristics and external validation sample/performance)
	20c	E	For model evaluation, show a comparison with the development data of the distribution of important	Page 7/ 12, Results, 3.1 and 3.6 (training/test lesion and image counts by outcome

TRIPOD+AI

Section/Topic	Item	Development / evaluation	Checklist item	Reported on page
**TITLE**				
			predictors (demographics, predictors, and outcome).	category; external validation counts by category)
Model development	21	D;E	Specify the number of participants and outcome events in each analysis (e.g., for model development, hyperparameter tuning, model evaluation)	Page 7-12, Results, 3.1, 3.3-3.6 and Tables 2-5 (training/test counts, physician comparison, inter-observer agreement, and external validation performance)
Model specification	22	D	Provide details of the full prediction model (e.g., formula, code, object, application programming interface) to allow predictions in new individuals and to enable third-party evaluation and implementation, including any restrictions to access or re-use (e.g., freely available, proprietary)	Page 4-6, Materials and Methods, 2.3-2.4;<br>Page 5, Figure 2 (model architecture/training details)
Model performance	23a	D;E	Report model performance estimates with confidence intervals, including for any key subgroups (e.g., sociodemographic). Consider plots to aid presentation.	Page 6-12, Results, 3.3-3.6; Tables 2-5; Figures 3 and 5 (internal test performance, physician performance, kappa agreement, and exploratory external validation)
	23b	D;E	If examined, report results of any heterogeneity in model performance across clusters. See TRIPOD Cluster for additional details.	Page 12, Exploratory external validation in a second institution reported in Results 3.6 and Table 5; no formal cluster-stratified performance or heterogeneity analysis was performed
Model updating	24	E	Report the results from any model updating, including the updated model and subsequent performance	Not applicable
**DISCUSSION**				
Interpretation	25	D;E	Give an overall interpretation of the main results, including issues of fairness in the context of the objectives and previous studies	Page 13-15, Discussion, Conclusions
Limitations	26	D;E	Discuss any limitations of the study (such as a non-representative sample, sample size, overfitting, missing data) and their effects on any biases, statistical uncertainty, and generalizability	Page 14-15, Discussion, Limitations paragraph (single-centre development, domain shift, small/imbalanced external validation cohort, selection bias, class imbalance, static images)
Usability of the model in the context of current care	27a	D	Describe how poor quality or unavailable input data (e.g., predictor values) should be assessed and handled when implementing the prediction model	Page 14-15, Discussion, Limitations;<br>Page 3-4, Materials and Methods, 2.1-2.2 (poor-quality images excluded; preprocessing described and applied to training, test, and validation images)
	27b	D	Specify whether users will be required to interact in the handling of the input data or use of the model, and what level of expertise is required of users	Page 4, Materials and Methods, 2.2 (physician image -only interpretation);<br>Page 15, Conclusions (image-level decision-support tool requiring clinical integration)
	27c	D;E	Discuss any next steps for future research, with a specific view to applicability and generalizability of the model	Page 14-15, Discussion, Limitations; Conclusions (larger multicenter, external, and prospective validation; standardized acquisition; subgroup analyses; cine clips and additional modalities)
